# Impact of Virtual Care With Remote Automated Monitoring on the Rate of Acute Hospital Care Post Discharge and Index Length of Hospital Stay: Protocol for the Post Discharge After Surgery Virtual Care With Remote Automated Monitoring Technology 3 (PVC-RAM-3) Trial

**DOI:** 10.2196/72672

**Published:** 2025-06-02

**Authors:** Sandra Ofori, Michael H McGillion, Flavia K Borges, Carley Ouellette, Ameen Patel, David Conen, Maura Marcucci, Michael Ke Wang, Lily Jaeyoung Park, Conor Bell, Jennifer Lounsbury, Kanae Nagatani, Vikas Tandon, Trevor J Wilkieson, Ahraaz Wyne, Valerie Harvey, Stephanie Harrison, Rahima Nenshi, Jessica Bogach, John Harlock, Margherita Cadeddu, Shawn Forbes, Shariq Haider, Reza D Mirza, Sunita Narang, Clare J Reade, Daniel M Tushinski, Amit Raut, Samir Raza, Ted Scott, Anthony Adili, Jeremy Petch, PJ Devereaux

**Affiliations:** 1 McMaster University Hamilton, ON Canada; 2 Hamilton Health Sciences Hamilton, ON Canada; 3 Population Health Research Institute Hamilton, ON Canada

**Keywords:** elective surgery, virtual care, remote automated monitoring, index length of stay, acute hospital care, randomized controlled trial

## Abstract

**Background:**

A substantial proportion of patients require acute hospital care after hospital discharge post surgery, and many regions and countries have surgical backlogs.

**Objective:**

The Post Discharge After Surgery Virtual Care with Remote Automated Monitoring Technology-3 (PCV-RAM-3) trial tests the hypothesis that informing surgeons and patients of virtual care with remote automated monitoring (VC-RAM) assignment will promote earlier discharge, thereby reducing the index length of hospital stay, and that postdischarge VC-RAM will reduce acute hospital care.

**Methods:**

The PVC-RAM-3 trial is a randomized controlled trial that compares VC-RAM to standard postdischarge care among 2500 adults undergoing elective noncardiac surgery in 3 Canadian hospitals. Following the randomization of patients prior to surgery, surgeons and patients are immediately notified whether the patient has been allocated to the VC-RAM or control group. Outcome adjudicators remain blinded to each participant’s group assignment. Patients in the intervention arm learn to use a Health Canada–approved cellular modem–enabled tablet computer and Bluetooth-enabled remote automated monitoring technology from Cloud DX to take daily wound photos for 7 days and measure daily vital signs (ie, blood pressure, heart rate, oxygen saturation, temperature, and weight) three times daily on days 1-7 and twice daily on days 8-14 post discharge, along with completing a brief recovery survey. Nurses review these data and conduct scheduled virtual visits (days 1, 3, 7, and 14). Nurses will escalate care to a preassigned and available perioperative care physician if predetermined vital sign thresholds are exceeded, concerning symptoms arise, or a medication error is detected. These physicians manage the issues and add or modify treatments as needed. The standard care group will receive postdischarge care as per the standard of care at the hospital where they undergo surgery. The coprimary outcomes are acute hospital care and the index hospital length of stay within the first 30 days after randomization.

**Results:**

Study recruitment and follow-up are completed, and analysis of the study results is underway.

**Conclusions:**

This trial will offer insights into the role of VC-RAM in reducing acute hospital care and index length of hospital stay among adults undergoing elective surgery.

**Trial Registration:**

ClinicalTrials.gov NCT05171569; https://clinicaltrials.gov/ct2/show/NCT05171569

**International Registered Report Identifier (IRRID):**

DERR1-10.2196/72672

## Introduction

Each year, more than 100 million adults (including >500,000 Canadians) aged 45 years and older undergo major surgery; of these, 29% develop prognostically important complications, 7.5% require hospital readmission, and >1.5% die within 30 days [1,2]. A Canadian Institutes of Health Information study analyzing 2.1 million acute hospitalizations found that surgical patients (both inpatient and same-day procedures) accounted for 31% of cases. Among the surgical cases, 7% had an unplanned 30-day readmission (costing CAD $9700 [US $6985.70] per readmission), and 19% returned to the emergency department within 30 days [3]. Overall, 20%-25% of adults discharged after elective surgery require acute hospital care within 30 days. With rising surgical volumes outpacing the growth of hospital bed capacity, surgical backlogs continue to grow [4,5]. Addressing these challenges requires innovative strategies to both shorten hospital length of stay (LOS) and reduce readmissions after surgery.

Virtual care with remote automated monitoring (VC-RAM) has emerged as a promising approach to improve postsurgical transitions to the home [6,7]. Virtual care encompasses all the ways that health care providers remotely interact (eg, telephone and tablet computer) with their patients. Remote automated monitoring (RAM) refers to the use of technology to remotely obtain data regarding patients’ biophysical parameters (eg, blood pressure) [6].

In our previous Post Discharge After Surgery Virtual Care with Remote Automated Monitoring Technology-1 (PVC-RAM-1) trial, 905 patients who had undergone nonelective surgery were randomized to VC-RAM for 30 days or standard care [8]. Patients in the experimental group used a cellular-enabled tablet and RAM technology that collected biophysical data, took wound photographs, and engaged in scheduled, and when needed, unscheduled virtual visits with nurses and physicians. There were threshold-based alerts that immediately triggered nurse-physician interventions to ensure the timely detection and management of abnormalities. We found a signal for ﻿the reduction in acute-hospital care (22% in the virtual care group vs 27.3% in the standard-care group; relative risk 0.80, 95% CI 0.64-1.01), brief acute hospital care (13.7% vs 18.1%; relative risk 0.75, 95% CI 0.56-1.02), hospital readmission (9.5% vs 12.8%; relative risk 0.77, 95% CI 0.53-1.11), and emergency department visits (19.7% vs 24.4%; relative risk 0.81, 95% CI 0.64-1.04) [8].

It is only credible to expect VC-RAM to impact outcomes if identified problems lead to a change in management. In PVC-RAM-1, centers could adjust the threshold parameters for care escalation. Post hoc analyses demonstrated that in the centers with the highest escalations of care (ie, centers where nurses identified, and physicians rapidly addressed abnormalities based on adherence to the predetermined vital sign thresholds), substantial absolute reductions in these outcomes were realized: acute-hospital care 14.1% (95% CI 5.2-23.0); brief acute-hospital care 11% (95% CI 3.6-18.4); and emergency department visits 14.2% (95% CI 5.4-23.0). However, these findings were post hoc and hypothesis-generating, requiring confirmation through an adequately powered trial. Additionally, the impact of advanced knowledge that a patient will receive VC-RAM on discharge timing and subsequent outcomes remains unclear.

The use of VC-RAM for patients discharged home after surgery can help identify and manage early postoperative complications. We hypothesize that this approach will reduce the need for acute hospital care, which includes hospital readmissions or emergency department visits after discharge. Additionally, we believe that if surgeons and patients know that postdischarge virtual care and RAM are available, they may be more inclined to opt for earlier discharges, potentially decreasing the length of the index hospital stay. To test these hypotheses, we are evaluating the impact of our VC-RAM intervention compared to standard postdischarge care on two coprimary outcomes: the rate of acute hospital care postdischarge and the index length of hospital stay.

By evaluating both acute hospital care and index LOS, this trial aims to build on lessons from PVC-RAM-1, extending to elective surgeries and providing evidence on integrating VC-RAM into routine postsurgical care. The goal is to optimize safe discharge and patient outcomes while addressing significant systemic challenges like bed capacity and surgical backlogs.

## Methods

### Design and Setting

**Figure 1:**
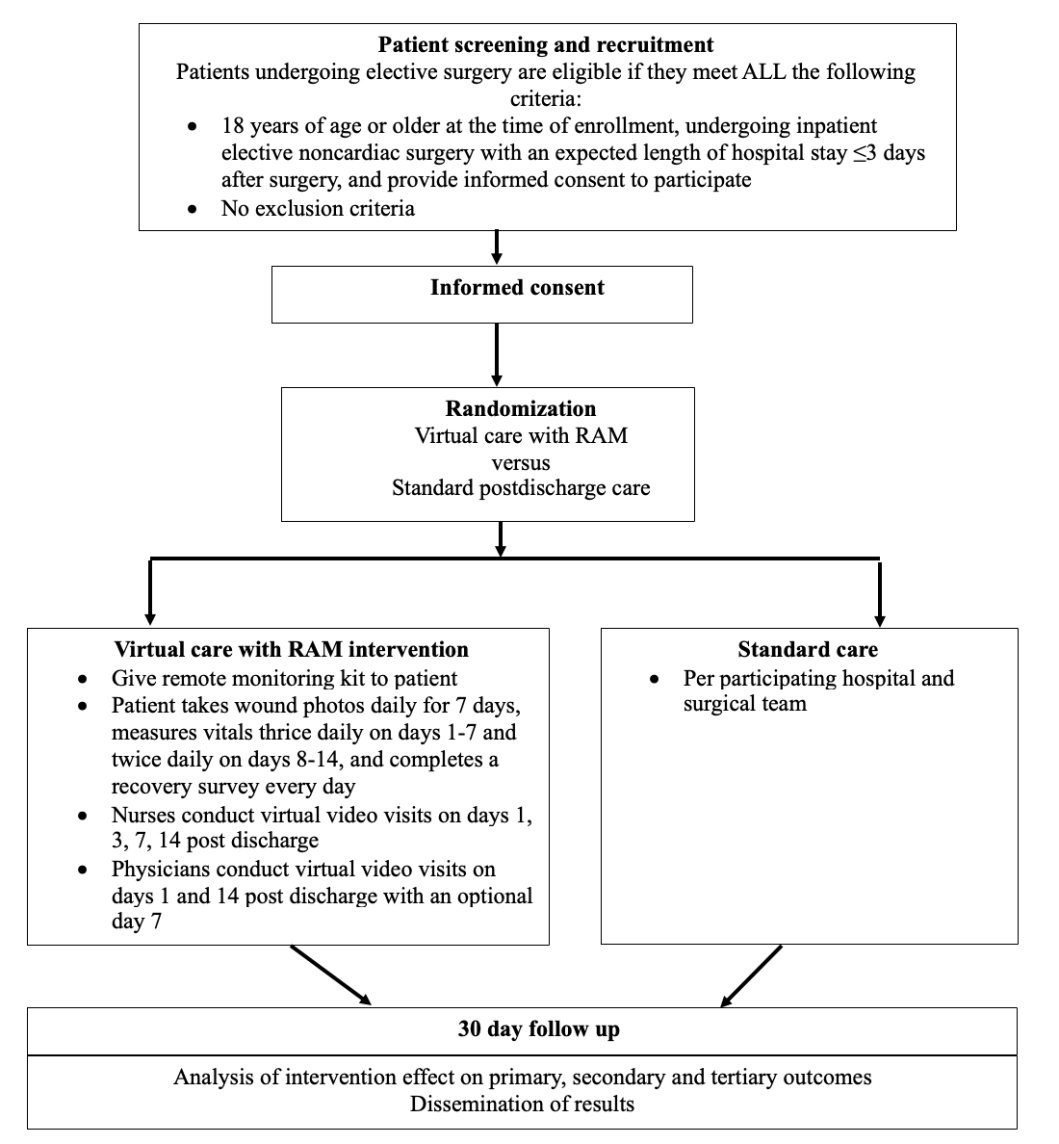
Trial flow diagram. RAM: remote automated monitoring.

The Post Discharge After Surgery Virtual Care with Remote Automated Monitoring Technology-3 (PVC-RAM-3) trial is a multicenter, parallel-group superiority randomized controlled trial involving 2500 patients undergoing elective noncardiac surgery at 3 Canadian hospitals: Hamilton General Hospital, Juravinski Hospital and Cancer Center, and St. Joseph’s Healthcare Hamilton. The trial evaluates the effects of VC-RAM compared to standard care post discharge. A study flow diagram is provided in Figure 1, and the SPIRIT (Standard Protocol Items: Recommendations for Interventional Trials) checklist is provided in Multimedia Appendix 1.

### Trial Population

Patients included in the trial are 18 years of age or older, undergoing inpatient elective noncardiac surgery with an expected hospital stay of ≤3 days after surgery, and provide informed consent. Exclusion criteria include the inability to communicate with research staff, complete study surveys, or undertake an interview using a tablet computer due to a language barrier or a cognitive, visual, or hearing impairment. Patients residing in areas without cellular network coverage are also excluded.

### Patient Recruitment

Study personnel use efficient recruitment strategies we previously developed in our perioperative trials, including identifying eligible patients through daily screening of surgical lists [8-10]. Surgeons with appropriate surgical populations (ie, surgeries with an anticipated LOS of 3 or fewer days) are contacted prior to any recruitment of their patients and asked to confirm their willingness to have their patients participate. Research personnel approach eligible patients during the preoperative period to obtain written informed consent (Multimedia Appendix 2).

### Randomization

Randomization occurs before surgery, after eligibility is confirmed, and informed consent is obtained. We randomize as close to the time of surgery as possible to minimize the risk of surgery postponement. Patients are randomized using an Interactive Web Randomization System, a 24-hour computerized system maintained by the coordinating center at the Population Health Research Institute, a joint institute of Hamilton Health Sciences and McMaster University in Hamilton, Ontario, Canada.

The randomization process uses block randomization with varying block sizes stratified by site, ensuring that study personnel and investigators remain unaware of block sizes. Patients are randomized in a 1:1 ratio to receive VC-RAM or standard care. Following randomization, research personnel notify the patient’s surgeon and the patient of the treatment allocation. Patients, health care providers, and data collectors are aware of treatment assignments, while outcome adjudicators remain blinded to treatment allocation.

### Trial Interventions

Patients are randomized to receive VC-RAM or standard care. Participating surgeons receive information on the trial protocol, objectives, and intervention model through rounds, in-service education, and discussions with trial researchers. This ensures that early discharge decisions for their patients are guided by their clinical judgment and understanding of the intervention care model (ie, when patients are assigned to the VC-RAM group, the patient will go home with VC-RAM to facilitate virtual care and vital sign monitoring).

**Figure 2:**
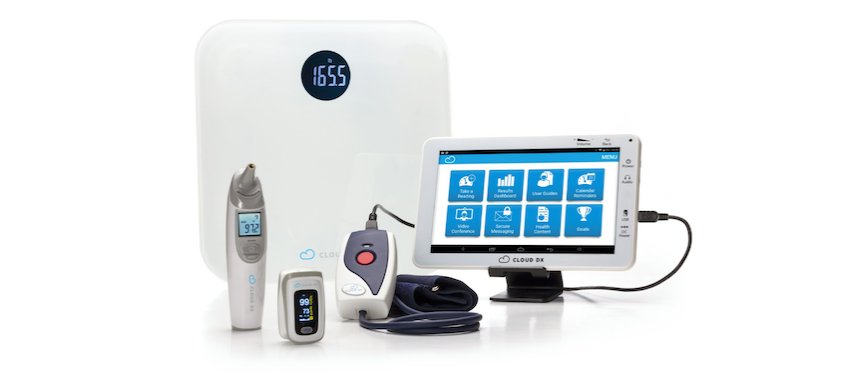
Cloud DX Connected Health kit. Cloud DX Connected Health kit, including Bluetooth-enabled Pulsewave wrist cuff blood pressure monitor, body weight scale, wireless oximeter, and temperature probe, paired with an Android health tablet. Reproduced with permission from Cloud DX.

The Cloud DX tablet is equipped with a camera and Bluetooth-enabled, Health Canada–licensed RAM technology (Figure 2). The Cloud DX Connected Health mobile app, hosted on the tablet, serves as the primary interface for the virtual care intervention and complies with the Personal Information Protection and Electronic Documents Act and the Personal Health Information Protection Act. This system has undergone extensive validation and user testing and was used in our previous PVC-RAM-1 trial [8,11]. The intervention begins the day after discharge and lasts 14 days or until 30 days post randomization, whichever comes first. The 14-day time frame is based on a period where major perioperative complications occur, and from our previous PVC-RAM-1 trial findings, where we determined that 30 days of monitoring often exceeds the needs of most patients [1,2,8]. Nurses establish contact and set up the system with patients as soon as possible after discharge, ensuring patients know how to seek help if they experience symptoms or issues arise.

Research staff teach patients (with family members participating where possible) in the VC-RAM group to use the automated Bluetooth-enabled monitoring devices paired with a preprogrammed, cellular modem–enabled tablet computer to measure their biophysical parameters, including blood pressure, heart rate, oxygen saturation, and temperature, three times daily on days 1-7 and twice daily on days 8-14. They measure their weight daily. They also complete a recovery survey developed for the trial, consisting of questions related to infection, bleeding, pain, dehydration, and cardiovascular and respiratory complications, daily. Patients photograph their wounds daily for the first 7 days unless otherwise directed. The biophysical parameters automatically upload via Bluetooth to the tablet, except for temperature, which is entered manually. These results are immediately wirelessly forwarded to the VC nursing team, and nurses actively review these results daily. If nurses do not receive a patient’s measurements, they will contact the patient at the earliest opportunity to understand the reasons behind the missing data. These vital signs, recovery surveys, and wound photos are parameters typically monitored in the hospital or during clinic visits, which we are now extending into the home setting.

Nurses conduct scheduled video visits on days 1, 3, 7, and 14 to assess symptoms, review wound photographs, reinforce recovery principles, and assess pain. They perform medication reconciliation on days 1, 7, and 14. Unscheduled video visits are arranged if biophysical measurements or survey responses exceed thresholds or if other health concerns are identified. Nurses escalate care to a preassigned physician when necessary, such as when RAM measurements exceed predetermined thresholds (examples in Multimedia Appendix 3), patients report specific symptoms (eg, shortness of breath), drug errors are identified, or concerns arise that require physician intervention. Physicians adjust treatments, order outpatient laboratory and diagnostic testing, liaise with surgeons, patients’ family physicians, or other health care providers, and arrange in-person clinic care when required.

Perioperative physicians hold scheduled video visits with patients on days 1 and 14 to assess patients, ensure proper medication use (by reviewing medication indications and proper dosing), and address medical needs. For patients who have a history of atherosclerotic disease, or are current smokers, an additional video call occurs on day 7 to optimize cardiovascular secondary prevention and smoking cessation therapy by ensuring that where indicated, patients were receiving secondary prevention medications (aspirin, statin, angiotensin-converting enzyme inhibitors/angiotensin II receptor blockers) and smoking cessation medications, respectively. These mechanisms ensure patients have 24/7 access to virtual nurses or physicians for urgent issues via secure video (Multimedia Appendix 4).

In both groups, discharge occurs when the most responsible surgeon deems it fit to discharge the patient. In the standard care group, patients receive postdischarge management according to the standard of care at the hospital where they underwent surgery. No changes to surgeons’ standard of care regarding postdischarge management occur for patients randomized to either group as a result of the trial. In Canada, standard postsurgery care typically involves follow-up with a health care provider within 30 days of discharge. Before this appointment, patients are responsible for contacting their surgeon or family physician with any concerns.

### Trial Outcomes

The PVC-RAM-1 trial provided encouraging data that virtual care with RAM may reduce acute hospital care [8]. In PVC-RAM-3, we wanted to inform the impact on acute hospital care and the index length of hospital stay. Initial sample size calculation assumptions suggested index length of hospital stay would require a larger sample size than acute hospital care. To avoid being underpowered by these outcomes, we made the index length of hospital stay the primary outcome. At the time of the final interim analysis, blinded overall data on the LOS and acute hospital care indicated there was adequate power to split the α and evaluate two coprimary outcomes (ie, acute hospital care and length of hospital stay). Consequently, we revised the primary outcome to include both index LOS and postdischarge acute hospital care as coprimary outcomes, making this change without any knowledge of the trial results.

The primary objective of this trial is to determine whether VC-RAM, compared with standard care after elective surgery, and informing surgeons and patients of VC-RAM allocation, reduces the occurrence of the coprimary outcomes within 30 days of randomization. The first coprimary outcome is postdischarge acute hospital care (defined as hospital readmission or emergency department visit) within 30 days after randomization. The second coprimary outcome is index hospital LOS (ie, time from the end of surgery to index hospital discharge).

Secondary outcomes during the first 30 days after randomization include (1) hospital readmission; (2) emergency department visit; (3) medication error detection; (4) medication error correction; (5) surgical site infection; (6) days in hospital; (7) pain of any severity, and moderate to severe pain assessed at 15 and 30 days after randomization; (8) proportion of patients with the atherosclerotic disease taking classes of efficacious medications at 30 days, (ie, an antiplatelet or anticoagulant drug, an angiotensin-converting enzyme inhibitor or angiotensin-receptor blocker, and a statin); and (9) the proportion of active smokers before surgery receiving pharmacological smoking cessation interventions at 30 days after randomization. Outcome definitions are presented in Multimedia Appendix 5.

Tertiary outcomes include (1) infection; (2) reoperation; (3) composite of myocardial infarction, acute heart failure, and arrhythmia that results in acute hospital care; (4) death; (5) health-related quality of life; and (6) health services usage–related costs.

We hypothesize that VC-RAM will reduce all primary, secondary, and tertiary outcomes, except for identifying more medication errors in the intervention group compared to the standard care group, which would reflect improved care through increased detection.

### Follow-Up

The day of randomization is day 1 of the trial, and all patients undergo 30 days of follow-up. Study personnel contact patients on day 30 to collect data on acute hospital care, secondary and tertiary outcomes, medication error detection and corrections, and current medications. The EQ-5D-5L is administered at baseline and day 30, and the Brief Pain Inventory-Short Form is administered on days 15 and 30. In the virtual care group, virtual nurses collect data on medication error detection and corrections throughout the intervention period.

If a patient reports an event, study personnel obtain source documentation from hospital or family physician records. If patients cannot be contacted directly, study personnel reach out to a designated relative, friend, or primary care physician. Study personnel and virtual nurses record all data in case report forms that are then entered into an electronic data capture system (Trial Master).

### Sample Size Calculations

Postdischarge acute hospital care and index hospital LOS are coprimary outcomes with a shared α. We allocate α=.04 for acute hospital care and α=.01 for index hospital LOS (details in Multimedia Appendix 6).

Table 1 presents the trial power for acute hospital care based on a log-rank test with a 2-sided α of .04 using SAS software (version 9.4; SAS Institute Inc). Assuming a 14% event rate in the control group, with an overall sample of size 2500 participants (1250 in the control group and 1250 in the treatment group), we will achieve 88.7% power to detect a relative risk reduction of at least 0.70 (30% relative risk reduction). Table 2 presents the trial power for index LOS based on a 2-sample Wilcoxon test assuming equal variance with a 2-sided α of .01. We expect patients in the standard care group to have a mean index hospital LOS of 2.5 days. If the knowledge that patients in the intervention group will receive virtual care with RAM after hospital discharge results in 35% of patients having their index hospital LOS reduced by 1 day, we will require a sample size of 2500 to have more than 80% power to detect this difference, with a 2-sided α of .01.

**Table 1:** Power to detect an RR^a^ (0.65, or 0.70, or 0.75) based on log-rank test with a 2-sided α of .04 assuming control group event rates 12%, 14%, 16%, and 18% for a given total sample of size 2500, 2800, 3000, or 3200.

Sample size per group			RR 0.65 (35% reduction)							RR 0.70 (30% reduction)							RR 0.75 (25% reduction)				
			Assumed control event rate (%)		Treatment P (%)		Power (%)		Assumed control event rate (%)			Treatment P (%)		Power (%)		Assumed control event rate (%)			Treatment P (%)		Power (%)
**2500**			12		7.8		—^b^		12			8.4		—		12			9		—
	1250	—		—		93.3		—			—		82.5		—			—		65.5	
	1400	—		—		95.6		—			—		86.7		—			—		70.6	
	1500	—		—		96.7		—			—		89.0		—			—		73.7	
	1600	—		—		97.6		—			—		90.1		—			—		76.5	
**2800**			14		9.1		—		14			9.8		—		14			10.5		—
	1250	—		—		96.6		—			—		88.7		—			—		73.4	
	1400	—		—		98		—			—		92		—			—		78.3	
	1500	—		—		98.6		—			—		93.6		—			—		81.1	
	1600	—		—		99		—			—		95		—			—		83.6	
**3000**			16		10.4		—		16			11.2		—		16			12		—
	1250	—		—		98.4		—			—		93		—			—		80	
	1400	—		—		99.1		—			—		95.4		—			—		84.4	
	1500	—		—		99.4		—			—		96.5		—			—		86.8	
	1600	—		—		99.6		—			—		97.4		—			—		88.9	
**3200**			18		11.7		—		18			12.6		—		18			13.5		—
	1250	—		—		99.3		—			—		95.8		—			—		85.3	
	1400	—		—		99.7		—			—		97.4		—			—		89.1	
	1500	—		—		99.8		—			—		98.2		—			—		91.1	
	1600	—		—		99.9		—			—		98.7		—			—		92.8	

^a^RR: relative risk.

^b^Not applicable.

**Table 2:** Power calculation based on 2-sample Wilcoxon tests assuming equal variance in LOSa with a 2-sided α of .01.

Standard care group expected index hospital LOS (days)	Intervention group expected impact on index hospital LOS	Sample size, n		
		80% power	85% power	90% power
3	40% of patients have their LOS reduced by 1.5 days	1078	1216	1400
3	35% of patients have their LOS reduced by 1.5 days	1450	1634	1882
3	30% of patients have their LOS reduced by 1.5 days	2030	2288	2634
3	40% of patients have their LOS reduced by 1.0 days	2618	2950	3396
3	35% of patients have their LOS reduced by 1.0 days	3482	3924	4518
3	30% of patients have their LOS reduced by 1.0 days	4826	5438	6260
2.5	40% of patients have their LOS reduced by 1.5 days	714	806	928
2.5	35% of patients have their LOS reduced by 1.5 days	968	1090	1256
2.5	30% of patients have their LOS reduced by 1.5 days	1362	1536	1768
2.5	40% of patients have their LOS reduced by 1.0 days	1764	1988	2290
2.5	35% of patients have their LOS reduced by 1.0 days	2356	2656	3058
2.5	30% of patients have their LOS reduced by 1.0 days	3280	3696	4254
2	40% of patients have their LOS reduced by 1.5 days	424	478	552
2	35% of patients have their LOS reduced by 1.5 days	582	656	754
2	30% of patients have their LOS reduced by 1.5 days	828	932	1074
2	40% of patients have their LOS reduced by 1.0 days	1078	1216	1400
2	35% of patients have their LOS reduced by 1.0 days	1450	1634	1882
2	30% of patients have their LOS reduced by 1.0 days	2030	2288	2634

^a^LOS: length of stay.

### Statistical Analyses

We will analyze all data according to a prespecified statistical analysis plan developed and finalized before any investigator is unblinded.

Following the intention-to-treat principle, we will analyze patients in the treatment groups to which they were randomized. For both coprimary outcomes, we will compare all patients allocated to the PVC-RAM-3 intervention to all patients allocated to standard care.

For coprimary 1 (composite of hospital readmission or emergency department visit), we will compute the time to event from the discharge date and plot the cumulative incidence curves between the two treatment groups. We will use a Cox regression model to obtain hazard ratios and 95% CIs. Any acute hospital care event that occurs before patients undergo their index surgery, in cases where surgery is postponed, will not be included in this outcome.

For coprimary 2 (index hospital LOS), we will calculate the hospital LOS for both groups and compare them with a 2-sample Wilcoxon rank sum test. Given the large sample size, the 2-sample Wilcoxon rank sum test will produce results that will be similar to the 2-sample *t* test.

For the coprimary outcome analyses, we will censor patients whose surgeries were canceled and not rescheduled within 30 days of randomization.

VC-RAM will be considered effective if either of the 2 coprimary efficacy analyses is significant. We will use a gatekeeping with a fallback procedure to analyze their statistical significance [12]. We will start with an overall α of .05, split between the two outcomes with a computed 2-sided *P* value less than an α of .04 for postdischarge acute hospital care and less than an α of .01 for index length of hospital stay. If the first coprimary outcome achieves significance at an α level of <.04, the α from this test will be added to the second coprimary outcome’s α (ie, the α will be .05). However, if the first coprimary outcome is not significant, the second coprimary outcome will be tested at an α level of <.01.

For secondary and tertiary outcomes, we will use the chi-square test for binary or categorical variables and the 2-sample *t* test for continuous variables. We will assess the effect of VC-RAM using log-binomial regression models and report the corresponding relative risks and 95% CIs. For continuous outcomes, we will evaluate the intervention effect using analysis of covariance regression models to obtain effect estimates and their 95% CIs. Depending on the frequency of time-to-event outcomes, we may use the log-rank test to assess differences between groups. The α level for all secondary and tertiary outcomes will be set at .05. For the secondary and tertiary outcomes, we will not adjust for multiple testing as we will be evaluating the consistency of effect, number of outcomes, and size of the effect to offer insights into the reliability of any statistically positive secondary or tertiary outcomes. Economic analysis (Multimedia Appendix 7) will be done in a separate protocol and publication.

### Subgroup Analyses

We will analyze subgroups based on weekdays of randomization: Mondays and Tuesdays versus Wednesdays, Thursdays, and Fridays. We hypothesize that the treatment effect will be less among patients randomized mid to late week as they may have longer stay because their postsurgical recovery is more likely to overlap with weekends where there may be operational delays in discharge, such as the absence of physiotherapists, that cannot be influenced by knowledge of VC-RAM allocation. To examine this, we will include interaction terms between group assignment and weekday randomization into the Cox regression model, with significant interaction inferred at a *P* value of <.05.

### Interim Analyses

Two interim efficacy analyses based on the primary outcome occurred when 50% and 75% of the patients had been followed for 30 days. The data monitoring committee (DMC) used the modified Haybittle-Peto rule of 4 SDs (α=.0000633) for analyses in the first half of the trial (including the first planned interim analysis) and 3.5 SDs (α=.0004653) for all analyses in the second half. For a finding of the treatment to be considered significant, these predefined boundaries had to have been exceeded in at least two consecutive analyses, 2 or more weeks apart. The interim analyses were conducted under protocol version 1.0 dated December 22, 2021, and therefore, did not take into consideration the changes made to the primary outcome in protocol version 2.0 dated January 25, 2025.

### Ethical Considerations

This trial is conducted in compliance with the protocol, the principles outlined in the Declaration of Helsinki, and Good Clinical Practice. The Hamilton Integrated Research Ethics Board provided its approval for the protocol and consent form (project ID 14365) before initiating the study. Research personnel obtain informed consent for each patient before randomization. All patient information is stored in a high-security computer system and kept strictly confidential. Participant confidentiality is further ensured by using identification code numbers to correspond with treatment data in computerized files. Medical information obtained during the trial is considered confidential and is not disclosed to third parties. However, this information may be shared with the patient’s personal physician or other appropriate medical personnel responsible for the patient’s care.

### Trial Organization

The Population Health Research Institute is the sponsor and coordinating center for this trial, overseeing trial organization, randomization scheme development, study database creation, data consistency checks, data analysis, and study center coordination. The trial structure includes a project office, operations committee, steering committee, DMC, site principal investigators, coinvestigators, and adjudication committee. The coprincipal investigators, project officers, program manager, and research coordinator are responsible for the activities of the Project Office. If safety concerns arise during the trial, the DMC Chair convenes a formal committee meeting. The DMC reviews all available data and relevant external studies before making recommendations to the Project Office Operations Committee. If termination is considered, the committee explores all alternatives before a decision is made. A detailed charter governs the DMC’s activities. Details about trial groups and investigators are provided in Multimedia Appendix 8.

## Results

The first patient was randomized on December 29, 2021, and recruitment of the target 2500 patients was completed on October 25, 2024. The DMC conducted the first and second scheduled interim analyses and recommended continuing the trial. The final version of the study protocol was submitted prior to the unblinding of the trial investigators. Some protocol amendments were implemented during the trial, with the most recent version finalized near the end of the study period. This paper is based on the most recent version of the study protocol (version 2.0, January 1, 2025). The data will be analyzed, and the study results will be published in a separate paper.

## Discussion

### Overview

Reducing postdischarge acute hospital care and index length of hospital stay remains a critical unmet need for surgical patients. We hypothesize that VC-RAM holds significant potential to address these challenges by lowering the incidence of these key outcomes. Building on the lessons learned from the PVC-RAM-1 trial, which established a scalable and effective model for VC-RAM, the PVC-RAM-3 trial adapts the intervention for elective surgical populations and examines how presurgical knowledge of VC-RAM allocation influences discharge decisions.

Throughout the last decade, there have been substantial changes in the duration of hospital admission for some surgical interventions. The relationship between the length of hospital stays and readmission rates is unclear, as some research indicates that shorter hospital stays are associated with higher readmission rates, while other studies suggest that longer stays may lead to more readmissions [2,13]. To the extent that shorter hospital stays may result in the need for more acute hospital care post discharge, this identifies a greater need for interventions to decrease acute hospital care post discharge, an objective that our VC-RAM intervention seeks to address. Our inclusion of patients undergoing elective surgery with an expected LOS of ≤3 days in this trial focus on a lower-risk population, where hospital discharge is less likely to be delayed by competing comorbidities. This targeted approach allows for a meaningful evaluation of VC-RAM’s ability to safely shorten hospital stays and optimize postdischarge care. Previous evidence has not identified significant safety risks associated with VC-RAM [8], supporting its feasibility for broader implementation.

With our VC-RAM intervention, when escalation triggers are met, nurses promptly escalate care to the perioperative physicians. These physicians can then take several actions depending on the patient’s needs, including continuing remote monitoring, conducting a video visit, referring the patient to an outpatient diagnostic facility, initiating or altering medications, or arranging follow-ups with themselves or nonstudy doctors, such as the surgeon or family physician. These measures are designed to manage complications within the community or through virtual care, thereby reducing the necessity for acute hospital visits.

PVC-RAM-3 trial is powered to detect a 30% relative risk reduction in acute hospital care and a 1-day reduction in index LOS in at least 35% of patients. With estimated baseline rates of 20%-25% for acute hospital care and an average hospital stay of 2.5 days [7], a 30% reduction could prevent 6-7.5 acute care visits per 100 patients. Postpandemic elective surgery volumes for those meeting PVC-RAM-3 inclusion criteria is approximately 4500 surgeries per year. If we reduce the LOS by a day for 30% of these elective surgeries, we could free up hospital beds and increase surgical volume by an additional 1350 cases per year to address surgical backlogs. This has the potential to generate substantial cost savings for the health care system.

Our previous trial demonstrated the potential of VC-RAM to improve other patient-important outcomes, such as the identification and correction of drug errors, pain management, and the medical optimization of patients, including those with atherosclerotic disease, for whom proven secondary prevention therapies exist, and those who smoke tobacco [8]. However, significant opportunities remain to further optimize pharmacological therapy to enhance long-term health outcomes, and these are evaluated in PVC-RAM-3.

### Strengths and Limitations

This is a large randomized controlled trial that evaluates the impact of knowledge of assignment to receive VC-RAM on index LOS and VC-RAM on postdischarge acute hospital care. The intervention builds on our prior VC-RAM studies and is optimized for efficiency by shortening the intervention period from 30 to 14 days, reducing wound photo duration to 7 days, and strictly enforcing vital signs' escalation thresholds across all centers. A potential limitation of this study is the burden of frequent monitoring and patient contacts, as well as the possibility that the resources required to deliver the intervention may offset in-hospital cost savings in settings outside our health system, thereby limiting generalizability. However, a separate economic evaluation is planned to address this issue.

### Dissemination

We will disseminate findings through presentations at national and international conferences, publications in high-impact peer-reviewed journals, and posts on the “Reducing Global Perioperative Risk” Resource Center, a global multimedia platform linked to Elsevier’s online readership.

### Conclusions

The results of the PVC-RAM-3 trial will provide critical insights into the role of VC-RAM in addressing current health system pressures and enhancing perioperative care globally.

## Appendix

Multimedia Appendix 1 SPIRIT (Standard Protocol Items: Recommendations for Interventional Trials) checklist.

Multimedia Appendix 2 Consent form.

Multimedia Appendix 3 Example of vital sign thresholds and recommend nurse and physician actions.

Multimedia Appendix 4 Additional details on nurse and physician actions.

Multimedia Appendix 5 Outcome Definitions.

Multimedia Appendix 6 Sample size.

Multimedia Appendix 7 Economic Analysis.

Multimedia Appendix 8 Trial Groups and Investigators.

## Abbreviations

DMC: data monitoring committee
LOS: length of stay
PVC-RAM-1: Post Discharge After Surgery Virtual Care with Remote Automated Monitoring Technology-1
PVC-RAM-3: Post Discharge After Surgery Virtual Care with Remote Automated Monitoring Technology-3
RAM: remote automated monitoring
SPIRIT: Standard Protocol Items: Recommendations for Interventional Trials
VC-RAM: virtual care with remote automated monitoring

## Data Availability

The Population Health Research Institute (PHRI) believes the dissemination of clinical research results is vital, and the sharing of data is important. The datasets generated during this study are not publicly available because PHRI prioritizes access to data analyses to researchers who have worked on the trial for a significant duration, have played substantial roles, and have participated in raising the funds to conduct the trial. PHRI balances the length of the research study and the intellectual and financial investments that made it possible with the need to allow wider access to the data collected. Data will be disclosed only upon request and approval of the proposed use of the data by a review committee. Data requests from other non–PVC-RAM-3 (Post Discharge After Surgery Virtual Care with Remote Automated Monitoring Technology-3) investigators will not be considered until 5 years after the close of the trial. All data requests should be made to the corresponding author.
