# Correction: “Impact of Virtual Care With Remote Automated Monitoring on the Rate of Acute Hospital Care Post Discharge and Index Length of Hospital Stay: Protocol for the Post Discharge After Surgery Virtual Care With Remote Automated Monitoring Technology 3 (PVC-RAM-3) Trial”

**DOI:** 10.2196/78893

**Published:** 2025-07-10

**Authors:** Sandra Ofori, Michael H McGillion, Flavia K Borges, Carley Ouellette, Ameen Patel, David Conen, Maura Marcucci, Michael Ke Wang, Lily Jaeyoung Park, Conor Bell, Jennifer Lounsbury, Kanae Nagatani, Vikas Tandon, Trevor J Wilkieson, Ahraaz Wyne, Valerie Harvey, Stephanie Harrison, Rahima Nenshi, Jessica Bogach, John Harlock, Margherita Cadeddu, Shawn Forbes, Shariq Haider, Reza D Mirza, Sunita Narang, Clare J Reade, Daniel M Tushinski, Amit Raut, Samir Raza, Ted Scott, Anthony Adili, Jeremy Petch, PJ Devereaux

**Affiliations:** 1 McMaster University Hamilton, ON Canada; 2 Hamilton Health Sciences Hamilton, ON Canada; 3 Population Health Research Institute Hamilton, ON Canada

## Abstract

A substantial proportion of patients require acute hospital care after hospital discharge post surgery, and many regions and countries have surgical backlogs. The Post Discharge After Surgery Virtual Care with Remote Automated Monitoring Technology-3 (PCV-RAM-3) trial tests the hypothesis that informing surgeons and patients of virtual care with remote automated monitoring (VC-RAM) assignment will promote earlier discharge, thereby reducing the index length of hospital stay, and that postdischarge VC-RAM will reduce acute hospital care. The PVC-RAM-3 trial is a randomized controlled trial that compares VC-RAM to standard postdischarge care among 2500 adults undergoing elective noncardiac surgery in 3 Canadian hospitals. Following the randomization of patients prior to surgery, surgeons and patients are immediately notified whether the patient has been allocated to the VC-RAM or control group. Outcome adjudicators remain blinded to each participant’s group assignment. Patients in the intervention arm learn to use a Health Canada–approved cellular modem–enabled tablet computer and Bluetooth-enabled remote automated monitoring technology from Cloud DX to take daily wound photos for 7 days and measure daily vital signs (ie, blood pressure, heart rate, oxygen saturation, temperature, and weight) three times daily on days 1-7 and twice daily on days 8-14 post discharge, along with completing a brief recovery survey. Nurses review these data and conduct scheduled virtual visits (days 1, 3, 7, and 14). Nurses will escalate care to a preassigned and available perioperative care physician if predetermined vital sign thresholds are exceeded, concerning symptoms arise, or a medication error is detected. These physicians manage the issues and add or modify treatments as needed. The standard care group will receive postdischarge care as per the standard of care at the hospital where they undergo surgery. The coprimary outcomes are acute hospital care and the index hospital length of stay within the first 30 days after randomization. Study recruitment and follow-up are completed, and analysis of the study results is underway. This trial will offer insights into the role of VC-RAM in reducing acute hospital care and index length of hospital stay among adults undergoing elective surgery. ClinicalTrials.gov NCT05171569; https://clinicaltrials.gov/ct2/show/NCT05171569

In “Impact of Virtual Care With Remote Automated Monitoring on the Rate of Acute Hospital Care Post Discharge and Index Length of Hospital Stay: Protocol for the Post Discharge After Surgery Virtual Care With Remote Automated Monitoring Technology 3 (PVC-RAM-3) Trial” (JMIR Res Protoc 2025;14:e72672) the authors made one correction to Figure 1. 

In the originally published version, the text in Figure 1 appeared as *“Patient takes wound photos daily for 7 days, measures vitals every day on days 1-7 and every other day on days 8-14, and complete recovery survey everyday”.* The original Figure is available in Multimedia Appendix 1.

The text in Figure 1 has been corrected to *“Patient takes wound photos daily for 7 days, measures vitals thrice daily on days 1-7 and twice daily on days 8-14, and completes a recovery survey every day”.*

**Figure 1:**
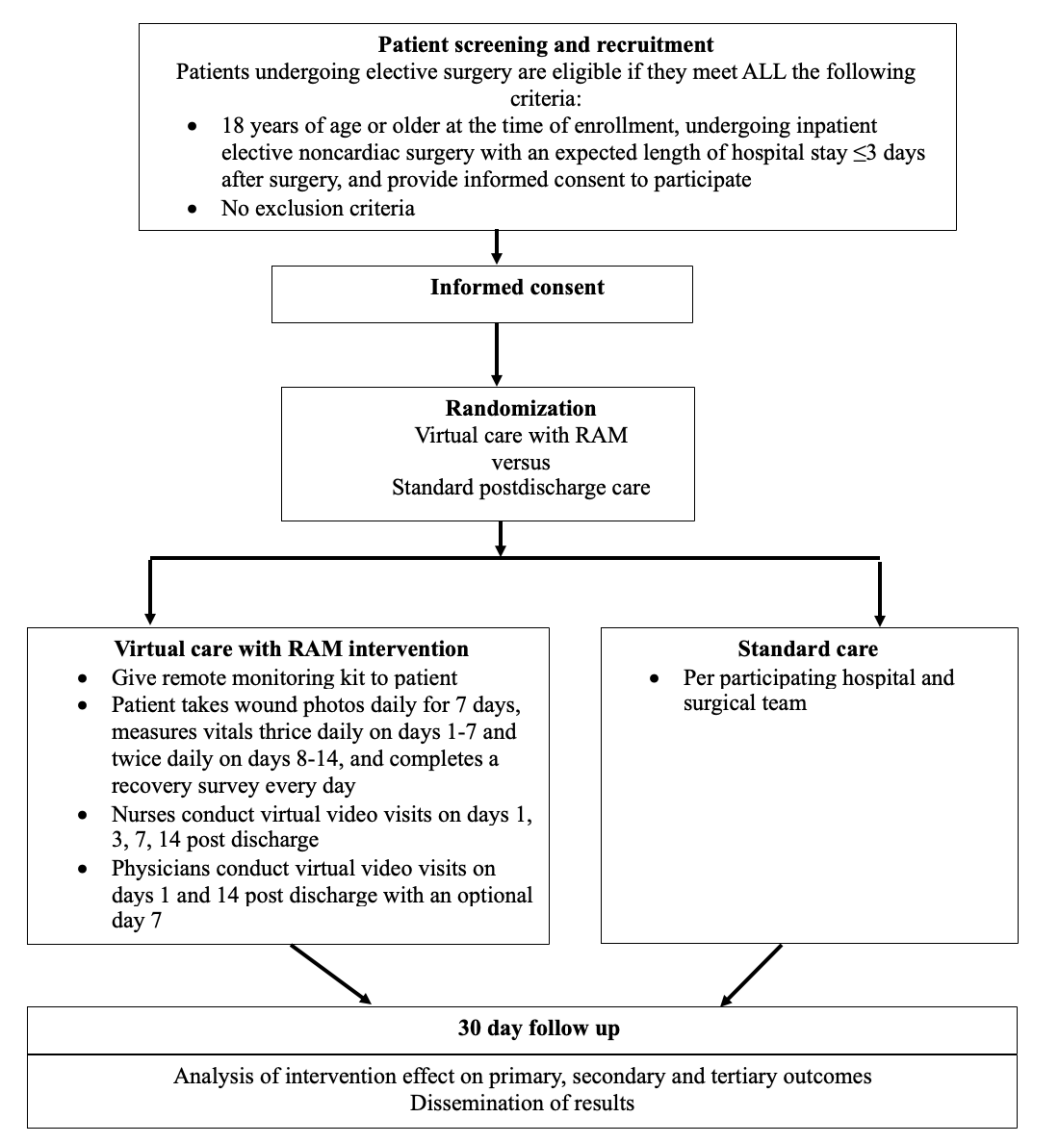
Trial flow diagram. RAM: remote automated monitoring.

The correction will appear in the online version of the paper on the JMIR Publications website together with the publication of this correction notice. Because this was made after submission to PubMed, PubMed Central, and other full-text repositories, the corrected article has also been resubmitted to those repositories.

## Appendix

Multimedia Appendix 1 Originally published version of Figure 1.

