# Supplementary material for: Impact of Virtual Care With Remote Automated Monitoring on the Rate of Acute Hospital Care Post Discharge and Index Length of Hospital Stay: Protocol for the Post Discharge After Surgery Virtual Care With Remote Automated Monitoring Technology 3 (PVC-RAM-3) Trial
Source: JMIR Res Protoc. 2025 Jun 2;14:e72672. doi: 10.2196/72672 (PMC12171644; doi:10.2196/72672)
Supplement: Multimedia Appendix 2 [file resprot_v14i1e72672_app2.docx]

**Informed Consent Form for Participation in a Research Study**

**Study Title**: Post discharge after surgery Virtual Care with Remote Automated Monitoring technology-3 (PVC-RAM-3) Trial

**Study Doctor**: *Dr. X*

Co-Investigator: *Dr. Y*

**Sponsor/Funder(s):** Population Health Research Institute (PHRI)

**INTRODUCTION**

You are being invited to participate in a clinical trial (a type of study that involves research). You are invited to participate in this trial because you are aged 18 or older and are scheduled to have non-cardiac surgery with an expected length of stay of 3 days or less. This consent form provides you with information to help you make an informed choice. Please read this document carefully and ask any questions you may have. All your questions should be answered to your satisfaction before you decide whether to participate in this research study.

The study staff will tell you about the study timelines for making your decision.

Taking part in this study is voluntary. You have the option to not participate at all or you may choose to leave the study at any time. Whatever you choose, it will not affect the usual medical care that you receive outside the study.

You will not be able to participate if you are unable to communicate with research staff, complete study surveys, or undertake an interview using a tablet computer due to a cognitive, language, visual, or hearing impairment; and/or reside in an area without cellular network coverage.

**IS THERE A CONFLICT OF INTEREST?**

There are no conflicts of interest to declare related to this study.

**WHAT IS THE BACKGROUND INFORMATION FOR THIS STUDY?**

With the current pandemic of COVID-19 and long surgery wait lists, it is important to safely reduce length of hospital stay after surgery while providing patients with access to treatment or care from a nurse or doctor. We need to evaluate better ways of managing people’s care after surgery to help prevent post surgery health problems and help people recover safely at home with access to nurses and doctors for medical care if needed.

Surgery is done to improve survival and quality of life. Unfortunately, complications like pain and infection can be a problem for some people during their recovery after surgery. Your risk of wound infection, hospital readmission, or need to go to an emergency department or urgent care centre, is greatest in the first couple of weeks following your surgery. Many complications, such as infection, pain, and medication errors can happen in the first few days following discharge home. If these problems are not dealt with fast enough, people can end up being readmitted to hospital or going to the emergency room. You may not see your surgeon, cardiologist, or family doctor for several days or weeks after surgery for your routine post-surgery follow up.

The standard or usual follow up timeline after surgery is a follow up visit or call from your surgeon. Depending on the type of surgery you have had, this follow up could be a telephone call or visit within a few days of your surgery, or up to 8 weeks after your surgery. During the COVID-19 pandemic, these timelines may be even longer.

**WHY IS THIS STUDY BEING DONE?**

The primary purpose of this study is to evaluate the planned use (patients and surgeons know this before surgery) of hospital-to-home care tools to reduce length of hospital stay and discharging patients from hospital earlier and monitoring recovery safely at home after surgery.

**WHAT OTHER CHOICES ARE THERE?**

You do not have to take part in this study in order to receive treatment or care. Currently, home monitoring is not part of the standard of care after surgery. Your doctor will provide the best care for you.

**HOW MANY PEOPLE WILL TAKE PART IN THIS STUDY?**

It is anticipated that about 2500 people will take part in this study from research sites located in Canada. This study should take one to two years to complete and the results should be known about 6 months after completion.

**WHAT WILL HAPPEN DURING THIS STUDY?**

**ASSIGNMENT TO A GROUP**

If you decide to participate then you will be "randomized" into one of the groups described below. Randomization means that you are put into a group by chance (like flipping a coin). There is no way to predict which group you will be assigned to. You will have an equal chance of being placed in either group. Neither you, the study staff, nor the study doctors can choose

what group you will be in. After randomization, you will be told which group you are in. Your healthcare team (e.g., surgeon), study doctors, study nurses, and research teams will also know which group you are in.

**WHAT IS THE STUDY INTERVENTION?**

**Group 1 (Virtual Care and remote monitoring):** Standard care (contact or visit with your surgeon after discharge and within the usual timeframe determined by your surgeon), plus virtual care and remote automated monitoring.

If you are randomized to this group you will be monitored at home by study nurses for 14 days after discharge. You will be provided with a hospital to home kit that contains the following technologies: tablet computer (with stand), wrist or upper arm blood pressure cuff (for blood pressure, pulse and breathing rate), finger worn pulse oximeter (for measuring your oxygen levels), thermometer (for temperature) and weigh scale (to monitor your weight). Monitoring will include video visits with a nurse on days 1, 3, 7, and 14 after discharge. These video visits will take approximately 15 minutes to start. As you recover and become more comfortable with the technologies, the visits may become shorter. You will also have a video visit with a perioperative physician on days 1 and 14. The physician will ensure you are taking the right medications and address any immediate medical needs.

Prior to discharge you will receive the hospital to home kit which will include instructions on how to set up the devices, and the planned 14-day monitoring schedule. You will review the kit and follow up schedule with research and/or hospital staff prior to or during your first video visit. You will be provided with training on how to use monitoring devices and the tablet application during the first call. Research staff will also answer any questions you have.

The measurements collected from the monitoring devices will be uploaded automatically to the companion tablet provided in the monitoring kit. The study nurse will then be able to review these measurements with you during your video visits. The frequency of daily vitals measurements will be 3 times a day for the first 7 days (morning, noon, and afternoon) and then twice a day (morning and afternoon) from day 8 until 14 days after you start the program. Weight will be measured daily in the morning before breakfast. It is expected that at least one full set of vitals will be recorded each day of the study. In addition to the vitals collection, each day, for 14 days, you will answer surveys about how you are feeling. Your answers will be uploaded to the study nurse for review and may be discussed with you during the video visit.

During the first 7 days of the program you will take a photograph of your wound, using the monitoring kit tablet, which will be securely sent to study nurses. The study nurses will review the photograph of your wound to look for signs of infection.

During the video visits, the study nurse will also ask you questions about your current medications, pain level, and address any other questions or concerns you may have about your recovery. In addition to nursing follow ups, the research team will contact you approximately 15 days after your surgery and ask you detailed questions about your level of pain. This call will be about 5 minutes long.

After the 14-day monitoring period has ended, you will be contacted by a member of the study team by phone at approximately 30 days after your surgery and asked a few questions about your overall health, current medications, any complications you had during the follow up period, any time spent in a hospital or other health care facility, any pain and your quality of life. You will be asked to package up the kit and prepare the kit for return at the end of the monitoring program.

**Group 2 (Standard Care):** Standard care (contact or visit with your surgeon after discharge and within the usual timeframe determined by your surgeon).

If you are randomized to this group you will receive the usual care provided for your recovery after surgery. You will be contacted by a study team member via telephone on approximately day 15 and 30 after your surgery. The phone call on day 15 will last approximately 5 minutes. During these calls, you will be asked about any pain you may be experiencing. During the 30 day call you will be asked about any medications you are taking, if you have recently been to a hospital or other healthcare facility, any complications that happened during your recovery, any pain you may be experiencing, and your quality of life. This call will take 15-20 minutes.

**WHAT ELSE DO I NEED TO KNOW ABOUT THE STUDY INTERVENTION?**

If at any point the study nurse feels your condition has worsened or thinks that you may have experienced a complication based on your vitals and video interview, the study nurse will have access to a study doctor up to 24 hours a day/ 7 days a week, to escalate your care. The study doctor will be able to assess you through telephone or video visit, and/or may make clinical decisions (e.g., add or modify any of your treatments, refer you to other specialists, etc.).

Outside of the regularly scheduled video visits you will also have access to a study nurse, up to 24 hours a day/ 7 days a week, should you feel that a change in your condition requires urgent contact with a healthcare provider.

The at home monitoring system from CloudDX used in the PVC-RAM-3 study has previously been approved by Health Canada. The monitoring system is not being tested in this study. We are investigating the clinical workflow and clinical and patient application of these approved devices as a method to potentially decrease length of hospital stay after surgery.

The PVC-RAM-3 intervention is not a replacement for emergency treatment of conditions that are immediately life-threatening. If you experience a medical emergency that you feel poses immediate and life-threatening danger to you, call 911. If, however, you feel you need urgent medical care, please call the PVC-RAM-3 virtual nursing station, as your first point of contact for assistance.

**WHAT ARE THE RESPONSIBILITIES OF STUDY PARTICIPANTS?**

If you choose to participate in this study, you will be expected to:

- Tell the study doctor about your current medical conditions;
- Tell the study doctor about all prescription and non-prescription medications and supplements, including vitamins and herbals, and check with the study doctor before starting, stopping or changing any of these.
- Tell the study doctor if you are thinking about participating in another research study
- Use the remote monitoring equipment as instructed
- Do not tamper with the equipment. You are responsible for taking care of the equipment.
- Complete daily surveys, vital signs measurements, and scheduled visits with the study nurse (if you are receiving the intervention)
- Return the monitoring kit and all devices at the end of the follow up period (if you are receiving the intervention)

**HOW LONG WILL PARTICIPANTS BE IN THE STUDY?**

The study intervention will last for about 14 days after discharge and total follow up is approximately 30 days after your surgery. The study team will collect information from your hospital chart relevant to your time in hospital for the surgery that made you eligible to participate in this trial. This may include items like your medical history prior to surgery, laboratory assessments performed as part of your routine care, details of your operation, and information on your recovery between surgery and hospital discharge. If you are admitted to another hospital for any reason or die from natural or other causes while participating in this study, your medical records will be requested in order to collect information relevant to your study participation. By signing this consent form, you are allowing such access.

No matter which group you are randomized to, and even if you stop the study intervention early, we would like to keep track of your health and any time spent in hospital to provide data on health system use and cost after surgery. Data on hospital re-admission, length of stay, and healthcare use will be obtained from the Integrated Decision Support (IDS) database. IDS uses information available in your electronic medical records for health system and community healthcare use in Ontario. Data in IDS is pre-linked using your health card number (HCN) and your data will be assigned a unique identifier in the IDS system. No names and addresses are available IDS. Your health card number, date of birth and postal code are collected by IDS for linking purposes only. No direct identifiers are within the shared dataset.

**CAN PARTICIPANTS CHOOSE TO LEAVE THE STUDY?**

You can choose to end your participation in this research (called withdrawal) at any time without having to provide a reason. If you choose to withdraw from the study, you are encouraged to contact the study doctor or study staff and discuss the level of withdrawal. Information that was recorded before you withdrew will be used by the researchers for the purposes of the study, but no information will be collected or sent to the sponsor after you withdraw your permission. For purposes of safety and study integrity, the research team will make every effort to re-contact you either directly or through a third party (e.g., contacting your family or private physician, review hospital records, available registries, or health care database) to determine your vital status and any hospital visits (e.g., emergency department) or readmissions at the end of your previously scheduled 30 day follow up.

**CAN PARTICIPATION IN THIS STUDY END EARLY?**

The study doctor may stop your participation in the study early, and without your consent, for reasons such as:

- You are unable to complete all required study procedures
- The Sponsor decides to stop the study
- The Regulatory Authority/ies (for example, Health Canada) or research ethics board withdraw permission for this study to continue

If this happens, it may mean that you would not receive the study intervention for the full period described in this consent form. If you are removed from this study, the study doctor will discuss the reasons with you.

**WHAT ARE THE RISKS OR HARMS OF PARTICIPATING IN THIS STUDY?**

If you are randomized to the intervention, your surgeon may decide to discharge you earlier because they know you will receive at home monitoring. Your surgeon will base their decision on your recovery and what is the most appropriate care for you. There is a small risk that you could have a complication related to earlier discharge. You will also be required to use a wrist or upper arm blood pressure monitor, and a wireless pulse oximeter. These devices may feel uncomfortable at times. You may discuss comfort of these devices with the virtual care nurse during your video visits.

**WHAT ARE THE BENEFITS OF PARTICIPATING IN THIS STUDY?**

If you are randomized to the Virtual care and remote monitoring arm of the study you may benefit from the increased monitoring. In our prior research, we found our approach to virtual care could help people stay out of hospital once discharged. In this current study, we are looking to determine if our approach to virtual care can also have an influence on length of hospital stay. Due to the increased monitoring, you will also have increased contact with study nurses compared to standard care.

If you are randomized to the standard care arm of the study you may benefit from medication reconciliation and review at day 30. Research team may identify and correct (with help from local study physicians) a medication error.

Your participation in this study may add to the medical knowledge about remote monitoring in patients after surgery.

**HOW WILL PARTICIPANT INFORMATION BE KEPT CONFIDENTIAL?**

If you decide to participate in this study, the study doctors and study staff will only collect the information they need for this study. Records identifying you at this centre will be kept confidential and, to the extent permitted by the applicable laws, will not be disclosed or made publicly available, except as described in this consent document.

Authorized representatives of the following organizations may look at your original (identifiable) medical/clinical study records at the site where these records are held, to check that the information collected for the study is correct and follows proper laws and guidelines.

- Population Health Research Institute, the Sponsor of this study
- The Hamilton Integrated Research Ethics Board HiREB
- This institution and affiliated sites, to oversee the conduct of research at this location

Information that is collected about you for the study (called study data) may also be sent to the organizations listed above. Your name, address, email, or other information that may directly identify you will not be used. The records received by these organizations may contain your participant code, sex, and date of birth.

**The following organizations will also receive study data:**

- Integrated Decision Support (IDS)
- CloudDX Diagnostics Inc., technology service provider for in home monitoring and video follow up

Studies involving humans sometimes collect information on race and ethnicity as well as other characteristics of individuals, like level of education, because these characteristics may influence how people respond to different interventions. Providing information on your race or ethnic origin and level of education is voluntary.

This study requires access to Decision Support (IDS) database for purposes of analyses on health care use and cost (e.g., hospitalizations, length of hospital stay, and emergency department visits).

IDS is a shared collaborative not for profit, integrated data repository tool stewarded by the Ontario Hospital Association and hosted by Hamilton Health sciences. Data is only available to organizations, like Hamilton Health Sciences, who have signed a data sharing agreement and service agreements with IDS. IDS does not store personal health information under these agreements and falls within the Personal Health Information Protection Act (PHIPA) requirements.

During the upload process, data is pre-linked using your health card number and patients are assigned a unique identifier which becomes the key to link your healthcare encounters in Ontario. No names or addresses are available in IDS. Your health card number, date of birth and postal code are used to create the link only and are not stored within the shared repository.

The study investigators will not be provided with your personal health information and data directly, but will be provided with a de-identified dataset which will include your data combined with data of other participants. The risks associated with allowing IDS to use your information are minimal, as the data at IDS is analyzed in large groups of participants so individuals are never identified and your personal health identifiers are never stored. All analyses to answer researcher questions will be performed on IDS computers by IDS advisors and researchers who will only see de-identified data. No individuals (the researchers or the IDS staff) will see the data while knowing who the participant is. IDS staff have no access to your personal health data or the organizations from which it is collected.

By providing your consent to participate you are agreeing that the study team can use data captured by IDS for analyses like length of hospital stay and use of healthcare to calculate costs after surgery.

If the results of this study are published, your identity will remain confidential. It is expected that the information collected during this study will be used in analyses and will be published/ presented to the scientific community at meetings and in journals. This information may also be used as part of a submission to regulatory authorities around the world to support the approval of the study intervention.

The at-home system is not connected to the hospital. The Connected Health mobile application was designed by Cloud DX. Hamilton Health Sciences has taken steps to ensure that your health information will be securely managed with applicable privacy laws. The CloudDX system will be receiving your personal data entered and it will be stored. This will include your biophysical measurements (vitals), survey responses, notes entered by study nurses, and photographs of your wound which you will take and upload to the CloudDX system for remote monitoring. CloudDX will have access to your de-identified data for internal research, development, and regulatory filings. The Cloud DX Connected Health platform is a secure remote patient monitoring solution with all cloud data residing on secure Microsoft Azure servers. The cloud data storage resides entirely in Canada on servers physically located in Toronto and Quebec (including all backup images), which meet PHIPA compliance for cloud services against ISO 27001 and SOC 2 certifications.  During the study, your personal health information will be visible to research team members located across the participating hospital sites, and on restricted access by Cloud DX personnel for technical support. To ensure cybersecurity and patient privacy, the Samsung tablet supports cellular communications through Personal Information Protection and Electronic Documents Act (PIPEDA) and Personal Health Information Protection Act (PHIPA)-compliant cloud infrastructure. Any data stored in the CloudDX cloud will be de-identified before exporting to our research database.

This study will use the CloudDX pulsewave platform to perform intervention/collect data, which is an externally hosted cloud-based service. A link to their privacy policy is available here (<https://www.clouddx.com/#/privacy>).

**Please note that whilst this service has been approved by the Hamilton Integrated Research Ethics Board for collecting data in this study, there is a small risk, as with any platform such as this, of data collected on external servers falling outside the control of the research team. Please talk to the researcher if you have any concerns.**

**WILL FAMILY DOCTORS/HEALTH CARE PROVIDERS KNOW WHO IS PARTICIPATING IN THIS STUDY?**

Your family doctor/health care provider may be informed that you are taking part in a study so that you can be provided with appropriate medical care. If you do not want your family doctor/health care provider to be informed, please discuss this with the study team.

**WILL information about this study BE available online?**

A description of this clinical trial will be available on *https://clinicaltrials.gov*. This website will not include information that can identify you. You can search this website at any time.

**WHAT IS THE COST TO PARTICIPANTS?**

The virtual care and remote monitoring technologies will be supplied at no charge while you take part in this study. Participation in this study will not involve any additional costs to you or your private health care insurance.

**ARE STUDY PARTICIPANTS PAID TO BE IN THIS STUDY?**

You will not be paid for taking part in this study. In the case of research-related side effects or injury, medical care will be provided by the study investigator, to help you recover from the injury or refer you for appropriate treatment. Costs for medical care that you might incur for injuries or illnesses that are not a direct result of research activities will not be covered by the study.

**WHAT ARE THE RIGHTS OF PARTICIPANTS IN A RESEARCH STUDY?**

You will be told, in a timely manner, about new information that may be relevant to your willingness to stay in this study.

You have the right to be informed of the results of this study once the entire study is complete. The results of this study will be available on the clinical trial registry (see the “Will information about this study be available online” section for more details).

Your rights to privacy are legally protected by federal and provincial laws that require safeguards to ensure that your privacy is respected.

By signing this form, you do not give up any of your legal rights against the study doctor, sponsor or involved institutions for compensation, nor does this form relieve the study doctor, sponsor or their agents of their legal and professional responsibilities.

You will be given a copy of this signed and dated consent form prior to participating in this study.

**WHAT IF RESEARCHERS DISCOVER SOMETHING ABOUT A RESEARCH PARTICIPANT?**

During the study, the researchers may learn something about you that they didn’t expect. For example, the researchers may find out that you have another medical condition that was not previously diagnosed. If any new clinically important information about your health is obtained as a result of your participation in this study, it may be shared with your family doctor at your discretion. Should a new medical condition be identified that requires treatment, your care will be directed by study doctors. See the “What else do I need to know about the study intervention?” section for more details.

**WHOM DO PARTICIPANTS CONTACT FOR QUESTIONS?**

If you have questions about taking part in this study, or if you suffer a research-related injury, you can talk to your study doctor, or the doctor who is in charge of the study at this institution.

That person is:

XXXX XXXX XXX-XXX-XXXX ext XXXX

Name Telephone

This study has been reviewed by the Hamilton Integrated Research Ethics Board (HIREB). The HIREB is responsible for ensuring that participants are informed of the risks associated with the research, and that participants are free to decide if participation is right for them. If you have any questions about your rights as a research participant, please call the Office of the Chair, Hamilton Integrated Research Ethics Board at 905.521.2100 x 42013.

SIGNATURES

- All of my questions have been answered,
- I understand the information within this informed consent form,
- I allow access to medical records and related personal health information as explained in this consent form,
- I do not give up any legal rights by signing this consent form,
- I understand that my family doctor/health care provider may be informed of study participation
- I agree to take part in this study.
- I give permission for study personnel to obtain my medical information regarding my treatment received in any clinic or doctor’s office or any healthcare facility that I visit (including the Niagara Health System), for research purposes, for 3 years from the date this consent is signed. ❑ **Yes** ❑ **No**

____________________________ ______________________ _________________

Signature of Participant PRINTED NAME Date/Time

____________________________ ______________________ _________________

Signature of Person Conducting PRINTED NAME & ROLE Date/Time

the Consent Discussion
