# Supplementary material for: Impact of Virtual Care With Remote Automated Monitoring on the Rate of Acute Hospital Care Post Discharge and Index Length of Hospital Stay: Protocol for the Post Discharge After Surgery Virtual Care With Remote Automated Monitoring Technology 3 (PVC-RAM-3) Trial
Source: JMIR Res Protoc. 2025 Jun 2;14:e72672. doi: 10.2196/72672 (PMC12171644; doi:10.2196/72672)
Supplement: Multimedia Appendix 3 [file resprot_v14i1e72672_app3.docx]

| SBP measurement | Flag to nurse on CloudDX Connected Health Dashboard | Nurse recommended action | Physician recommended action |
| --- | --- | --- | --- |
| 100-115 mm Hg | Mild | Nurse to contact and assess patient during scheduled video call.  Advise patient to re-check measurement.  Nurse to update perioperative care physician, at daily rounds. | Rule out precipitating factors (e.g. sepsis, volume depletion, bleeding, heart failure).  Review medication and fluid intake.  Decrease blood pressure medication dosage accordingly.  Order back to nurse and coordinate follow up with nurse.  Reassess in 24 hours. |
| 86-99 mm Hg | Medium | Nurse to contact and assess patient within 30 minutes.  Advise patient to re-check measurement.  If unresolved, nurse will inform perioperative care physician within 1 hour. | All of the above and the following.  Withhold anti-hypertensives until SBP >100 mmHg if patient with no HFrEF. Assess volume status.  Order back to nurse and coordinate follow up.  Reassess in 4-6 hours |
| <85 mm Hg | High | Contact and assist patient immediately.  Advise patient to re-check measurement.  If unresolved, nurse will inform perioperative care physician within 15 minutes. | Assess patient for symptoms.  If patient is asymptomatic then all of the above and the following.  Withhold anti-hypertensives.  Consider video call with patient.  Consider clinic assessment.  Order back to nurse and coordinate follow up  If patient is symptomatic then all of the above and consider emergency room assessment. |

**Heart Rate – Tachycardia**

| **Rule** | **Flag** | **Nurse Action** | **Physician Action** |
| --- | --- | --- | --- |
| 100-119bmp | Mild | Nurse to monitor dashboard, monitor health trends, and if needed, contact and assess patient through video call  Advise patient to re-check measurement  Discuss with MRP as needed, based on assessment/ update at daily rounds | Nurse to update MRP at rounds |
| 120-149bpm | Medium | Contact and assess patient within 30 mins  Advise patient to re-check measurement  If unresolved, contact MRP within 1 hour | Nurse to contact MRP within 1 hour |
| >150bpm | High | Contact and assess patient immediately  Advise patient to re-check measurement  Contact MRP within 15 minutes | Nurse to contact MRP within 15 minutes |

**Heart Rate – Bradycardia**

| **Rule** | **Flag** | **Nurse Action** | **Physician Action** | |
| --- | --- | --- | --- | --- |
| 40-55bmp | Mild | Nurse to monitor dashboard, monitor health trends, and if needed, contact and assess patient through video call  Advise patient to re-check measurement  Discuss with MRP as needed, based on assessment/ update at daily rounds | | Nurse to update MRP at rounds |
| <40bpm | Medium | Contact and assess patient within 30 mins  Advise patient to re-check measurement  Contact MRP within 1 hour | | Nurse to contact MRP within 1 hour |

**SpO2 - Desaturation**

| **Rule** | **Flag** | **Nurse Action** | **Physician Action** |
| --- | --- | --- | --- |
| O2 Saturation reading:  90%-92% | Mild | Nurse to monitor dashboard, monitor health trends, and if needed, contact and assess patient through video call | No immediate action  Nurse will update MRP at rounds |
|  |  | Advise patient to re-check measurement | If daily rounds have been completed,  the nurse will contact MRP by the end of shift |
|  |  |  |  |
| O2 Saturation reading:  88%-89% | Medium | Nurse to contact and assess patient within 2 hours of reading | Nurse will update MRP at rounds |
|  |  | Advise patient to re-check measurement |  |
|  |  | Discuss with MRP within the day if ≥88%- ≤89% update MRP at rounds |  |
| O2 Saturation reading:  <88% | High | Contact and assess patient within 15-30 mins | Nurse to contact MRP within 1 hour |
|  |  | Advise patient to re-check measurement |  |
|  |  | If unresolved, contact MRP within 1 hour |  |

**SpO2 – for patients with COPD**

| **Rule** | **Flag** | **Nurse Action** | **Physician Action** |
| --- | --- | --- | --- |
| O2 Saturation reading:  88%-90% | Mild | Nurse to monitor dashboard, monitor health trends, and if needed, contact and assess patient through video call | No immediate action  Nurse will update MRP at rounds as required |
|  |  | Advise patient to re-check measurement |  |
|  |  |  |  |
| O2 Saturation reading:  <88% | High | Contact and assess patient within 15-30 mins | Nurse to contact MRP within 1 hour |
|  |  | Advise patient to re-check measurement |  |
|  |  | If unresolved, contact MRP within 1 hour |  |
