# Supplementary material for: Impact of Virtual Care With Remote Automated Monitoring on the Rate of Acute Hospital Care Post Discharge and Index Length of Hospital Stay: Protocol for the Post Discharge After Surgery Virtual Care With Remote Automated Monitoring Technology 3 (PVC-RAM-3) Trial
Source: JMIR Res Protoc. 2025 Jun 2;14:e72672. doi: 10.2196/72672 (PMC12171644; doi:10.2196/72672)
Supplement: Multimedia Appendix 4 [file resprot_v14i1e72672_app4.docx]

Before the trial commenced, trial nurses and perioperative physicians received standardized training in virtual care tailored to their roles in VC-RAM and the use of the clinician dashboard interface. Additionally, we developed a comprehensive manual to systematically standardize the postoperative review of patients and manage potential symptoms and complications.

During nurse visits, medication reconciliation is undertaken to identify any drug errors, including mistakes in prescribing, transcribing, dispensing, administering, or monitoring, due to preventable events or actions taken by a patient, caregiver, or healthcare worker. This process involves nurses reviewing all medications reported by the patient and reconciling these against pharmacy records and the patient’s after-visit summary, which is printed from the hospital's electronic medical records at discharge.

Our perioperative team consists of a multidisciplinary group of perioperative nurses and physicians (i.e., internists, cardiologists, hospitalists, anesthesiologists). One of these physicians and a nurse are available virtually 24/7, on a weekly schedule to ensure continuous patient support. Each scheduled video visit with a nurse or physician is planned for 30 minutes, although the actual duration may vary based on clinical needs.
