# Supplementary material for: Impact of Virtual Care With Remote Automated Monitoring on the Rate of Acute Hospital Care Post Discharge and Index Length of Hospital Stay: Protocol for the Post Discharge After Surgery Virtual Care With Remote Automated Monitoring Technology 3 (PVC-RAM-3) Trial
Source: JMIR Res Protoc. 2025 Jun 2;14:e72672. doi: 10.2196/72672 (PMC12171644; doi:10.2196/72672)
Supplement: Multimedia Appendix 5 [file resprot_v14i1e72672_app5.docx]

| **Outcome** | **Definition** |
| --- | --- |
| Index Length of Hospital Stay | Length of stay from end of surgery until discharge from the index hospitalization (measured in days and hours) |
| Acute-hospital care | Acute-hospital care is a composite outcome of hospital re-admission and emergency department visit, which includes urgent-care centre visit. |
| Hospital re-admission | Patient admission to an acute-care hospital. |
| Emergency department visit | Patient visit to an emergency department, which includes urgent-care centre visit. |
| Medication error detection | Medication errors include mistakes in medication prescribing, transcribing, dispensing, administering, or monitoring due to preventable events or actions taken by a patient, caregiver, or healthcare worker. Medication errors include: drug omission (i.e., patient did not take a drug they were supposed to take), drug commission (i.e., patient taking a drug they were not supposed to take), duration error, dosing error, frequency error, route error, and timing error. We will record all drug errors identified. |
| Medication error correction | Any medication error that is corrected. |
| Surgical site infection | Surgical site infection is an infection that occurs within 30 days after randomization and involves the skin, subcutaneous tissue of the incision (superficial incisional), or the deep soft tissue (e.g., fascia, muscle) of the incision (deep incisional). |
| Days in Hospital | Total number of days in hospital from randomization to 30 days post-randomization, including index hospital stay and any hospital readmission (s). A day in hospital is counted if a participant spends 1 minute of any day admitted to hospital. |
| Pain | Pain intensity and related interference with usual daily activities, will be measured via the Brief Pain Inventory-Short Form (BPI-SF).^4^ The BPI-SF includes four 11-point numeric rating scales (NRS) of pain intensity, which measure “average”, “least”, and “worst” pain intensity in the past 24 hours (hrs.), respectively, as well as pain intensity “now” (0= no pain, 10= pain as bad as you can imagine). The BPI-SF interference subscale will also be used, which measures the degree to which pain interferes with general activity, mood, walking, work, relations with others, sleep, and enjoyment of life (NRS for each item; 0=does not interfere, 10=completely interferes). A total interference score is determined by calculating the sum of these 7 items. The BPI-SF has strong psychometric properties with well-established reliability and validity across divergent surgical groups. Moderate to severe pain is defined by a score of ≥4/10 on a standard numeric rating scale (NRS) for pain. |
| Optimal pharmacological management among patients with atherosclerotic disease | Among patients with atherosclerotic disease, we will also assess optimal pharmacological management based upon whether patients are taking 0, 1, 2, or 3 of the following classes of efficacious medications at 30 days (i.e., an antiplatelet or anticoagulant drug; an angiotensin-converting enzyme inhibitor or angiotensin-receptor blocker; and a statin). Among active smokers before surgery we will assess if patients are receiving pharmacological smoking cessation interventions at 30 days after randomization. |
| Health services utilization-related costs | Data on hospital re-admission, length of stay, and healthcare utilization will be obtained from the Ontario Hospital Association Integrated Decision Support (IDS) database. IDS is a comprehensive platform with an integrated view of patient activity, allowing for access to health systems data for any patients enrolled. Data on costs of health service utilization will be obtained from the Ontario Case Costing Database. |
| Health-related quality of life (HRQoL) | HRQoL will be measured with the EQ-5D-5L instrument (https://euroqol.org/eq-5d-instruments/sample-demo) due to its increased sensitivity and validation in several countries including Canada.^4^  We use the EQ-5D-5L as it is recommended in Canada^5^ to calculate the Quality Adjusted Life Years (QALYs) when conducting cost-effectiveness analyses. It has broad applicability beyond disease specific instruments as our population includes a broad category of patients undergoing surgery. |
| Infection | Infection is defined as a pathologic process caused by the invasion of normally sterile tissue, fluid, or body cavity by pathogenic or potentially pathogenic organisms. |
| Re-operation | Re-operation refers to any surgical procedure undertaken for any reason (e.g., wound dehiscence, infection) that is related to the index surgery. |
| Myocardial infarction | The diagnosis of myocardial infarction requires one of the following criteria:   - - - 1. Detection of a rise or fall of a cardiac biomarker (preferably troponin) with at least one value above the 99^th^ percentile of the upper reference limit (URL) together with evidence of myocardial ischemia with at least one of the following:          1. ischemic signs or symptoms (i.e., chest, arm, neck, or jaw discomfort; shortness of breath, pulmonary edema);          2. development of pathologic Q waves present in any two contiguous leads that are ≥ 30 milliseconds;          3. new or presumed ECG changes indicative of ischemia (i.e., ST segment elevation [≥ 2 mm in leads V_1_, V_2_, or V_3_ OR ≥ 1 mm in the other leads], ST segment depression [≥ 1 mm], or symmetric inversion of T waves ≥ 1 mm) in at least two contiguous leads;          4. new LBBB; or          5. new cardiac wall motion abnormality on echocardiography or new fixed defect on radionuclide imaging          6. identification of intracoronary thrombus on angiography or autopsy       2. Cardiac death, with symptoms suggestive of myocardial ischemia and presumed new ischemic ECG changes or new LBBB, but death occurred before cardiac biomarkers were obtained, or before cardiac biomarker values would be increased.       3. Percutaneous coronary intervention (PCI) related myocardial infarction is defined by elevation of a troponin value (>5 x 99th percentile URL) in patients with a normal baseline troponin value (≤99th percentile URL) or a rise of a troponin measurement >20% if the baseline values are elevated and are stable or falling. In addition, either (i) symptoms suggestive of myocardial ischemia or (ii) new ischemic ECG changes or (iii) angiographic findings consistent with a procedural complication or (iv) imaging demonstration of new loss of viable myocardium or new regional wall motion abnormality are required.      - - - 1. Stent thrombosis associated with myocardial infarction when detected by coronary angiography or autopsy in the setting of myocardial ischemia and with a rise and/or fall of cardiac biomarker values with at least one of value above the 99th percentile URL.       2. Coronary artery bypass grafting (CABG) related myocardial infarction is defined by elevation of cardiac biomarker values (>10 x 99th percentile URL) in patients with a normal baseline troponin value (≤99th percentile URL). In addition, either (i) new pathological Q waves or new LBBB, or (ii) angiographic documented new graft or new native coronary artery occlusion, or (iii) imaging evidence of new loss of viable myocardium or new regional wall motion abnormality.       3. For patients who are believed to have suffered a myocardial infarction within 28 days of a MINS event or within 28 days of a prior myocardial infarction, the following criterion for myocardial infarction is required:   Detection of a rise or fall of a cardiac biomarker (preferably troponin) with at least one value above the 99^th^ percentile of the upper reference limit (URL) and 20% higher than the last troponin measurement related to the preceding event together with evidence of myocardial ischemia with at least one of the following:   - - - - 1. ischemic signs or symptoms (i.e., chest, arm, neck, or jaw discomfort; shortness of breath, pulmonary edema);         2. development of pathologic Q waves present in any two contiguous leads that are > 30 milliseconds;         3. new or presumed new ECG changes indicative of ischemia (i.e., ST segment elevation [> 2 mm in leads V_1_, V_2_, or V_3_ OR > 1 mm in the other leads], ST segment depression [> 1 mm], or symmetric inversion of T waves > 1 mm) in at least two contiguous leads;         4. new LBBB; or         5. new cardiac wall motion abnormality on echocardiography or new fixed defect on radionuclide imaging         6. identification of intracoronary thrombus on angiography or autopsy |
| Acute heart failure | The definition of acute heart failure requires at least one of the following clinical signs (i.e., elevated jugular venous pressure, respiratory rales or crackles, crepitations, or presence of S3) with at least one of the following:  1. radiographic findings of vascular redistribution, interstitial pulmonary edema, or frank alveolar pulmonary edema, OR  2. heart failure treatment with a diuretic and documented clinical improvement. |
| Arrhythmia that results in patient presenting to an emergency department or being admitted to hospital | Any arrhythmia that results in patient presenting to an emergency department, which includes an urgent care centre visit or being admitted to hospital. |
| Death | The definition of death is all cause mortality. |
