# Supplementary material for: Impact of Virtual Care With Remote Automated Monitoring on the Rate of Acute Hospital Care Post Discharge and Index Length of Hospital Stay: Protocol for the Post Discharge After Surgery Virtual Care With Remote Automated Monitoring Technology 3 (PVC-RAM-3) Trial
Source: JMIR Res Protoc. 2025 Jun 2;14:e72672. doi: 10.2196/72672 (PMC12171644; doi:10.2196/72672)
Supplement: Multimedia Appendix 6 [file resprot_v14i1e72672_app6.docx]

When we initially designed PVC-RAM-3, the sample size was prospectively based upon the original primary outcome of index hospital length of stay. We determined that enrollment of 2500 patients would give the trial >90% power to detect that virtual care with remote automated monitoring (RAM) will result in 35% of patients having their index hospital length of stay reduced by 1.0 day, at a two-sided alpha level of 0.05, on the assumption that the index hospital length of stay in the standard-care group would be 3 days.

Based on blinded overall data from the last Data Monitoring Committee report (with a sample size of 2347), the overall mean index hospital length of stay from the end of surgery to hospital discharge was 2.4 days (SD 3.1), and the overall proportion of hospital readmission or emergency department visits was 10.8%. Given that our data showed we had excessive statistical power to assess our original primary hypothesis, we revised the protocol to incorporate our main secondary outcome into the primary hypothesis treating "acute hospital care (i.e., hospital readmission or emergency department visit") and "index hospital length of stay" as co-primary outcomes, sharing the alpha. Specifically, we allocated an alpha of 0.04 for "Co-primary-1: “acute hospital care (i.e., hospital readmission or emergency department visit" and an alpha of 0.01 for "Co-primary-2: index hospital length of stay."

With our original sample size of 2500 (1250 per group), assuming a 30% relative risk reduction of hospital readmission and emergency department visit, we will have approximately 89% power for Co-primary-1 at a 4% level of significance. Assuming an average LOS of 2.5 days, we also have more than 80% power for Co-primary-2 at a 1% level of significance with 35% of patients having their LOS reduced by 1 day. This enhances our ability to comprehensively assess the effects of our intervention on both co-primary outcomes.
