# Supplementary material for: Impact of Virtual Care With Remote Automated Monitoring on the Rate of Acute Hospital Care Post Discharge and Index Length of Hospital Stay: Protocol for the Post Discharge After Surgery Virtual Care With Remote Automated Monitoring Technology 3 (PVC-RAM-3) Trial
Source: JMIR Res Protoc. 2025 Jun 2;14:e72672. doi: 10.2196/72672 (PMC12171644; doi:10.2196/72672)
Supplement: Multimedia Appendix 7 [file resprot_v14i1e72672_app7.docx]

We will conduct economic analyses to assess the costs and quality-adjusted life years (QALY’s) associated with virtual care with RAM and standard care. The costs of providing virtual care with RAM (e.g., start-up costs and provider time) will be derived from trial data. Healthcare resource utilization collected in the trial (e.g., hospitalizations, ED visits) will be costed using publicly available Canadian unit costs. Using the Canadian algorithm, the health utility scores derived from the EQ-5D-5L will be weighted by time spent in health states using an area-under-the-curve approach to calculate QALYs. Bootstrap techniques will be used to deal with sampling uncertainty and cost-effectiveness acceptability curves will be used to represent the uncertainty. The economic evaluation will be conducted according to Canadian and international guidelines and from a Ministry of Health perspective. The trial economic results will be used to inform the cost implications for the wider-Canadian setting.
