# Supplementary material for: Impact of Virtual Care With Remote Automated Monitoring on the Rate of Acute Hospital Care Post Discharge and Index Length of Hospital Stay: Protocol for the Post Discharge After Surgery Virtual Care With Remote Automated Monitoring Technology 3 (PVC-RAM-3) Trial
Source: JMIR Res Protoc. 2025 Jun 2;14:e72672. doi: 10.2196/72672 (PMC12171644; doi:10.2196/72672)
Supplement: Multimedia Appendix 8 [file resprot_v14i1e72672_app8.docx]

**Supplemental Trial Groups and Investigators**

**Participating centres and clinicians involved in the trial –** *Hamilton Health Sciences, Juravinski Hospital and Cancer Centre*: Amna Ahmed, Baha Alazzoni, Naif Alghamdi, Amitabha Chakroborty, Conor Cox, Emily Gregus, Amanda Huynh, Jessica Huynh, Kelly Lawrence; *Hamilton General Hospital*: Abdul Abulaban, Krysten Gregus, Kristen Lombardo, Shervin Zandi; *St. Joseph’s Healthcare Hamilton*: Eric Duan, Jacquie Hare.

**Population Health Research Institute Coordinating Centre:** Kumar Balasubramanian, Lori Blake, Eric Deng, Stephanie Harrison (Coordinator), Valerie Harvey, Shofiqul Islam, Peter Koh, Rajibul Mian, Natalie Ricotta, Jessica Vincent, Heidi Wilton.

**Event Adjudication Committee**: Flavia K Borges (Chair), Maura Marcucci (Chair), Wonho Kim, Francesca Mulazzani, Pavel Roshanov.

**Data Monitoring Committee:** Dan Sessler (Chair), Finlay McAlister, Kristian Thorlund. The members of the Data Monitoring Committee had expertise in clinical trials, perioperative care, virtual care, and statistics.
