# Supplementary figures and images for: Correction: “Impact of Virtual Care With Remote Automated Monitoring on the Rate of Acute Hospital Care Post Discharge and Index Length of Hospital Stay: Protocol for the Post Discharge After Surgery Virtual Care With Remote Automated Monitoring Technology 3 (PVC-RAM-3) Trial”
Source: J Med Internet Res. 2025 Jul 10;27:e78893. doi: 10.2196/78893 (PMC12292025; doi:10.2196/78893)

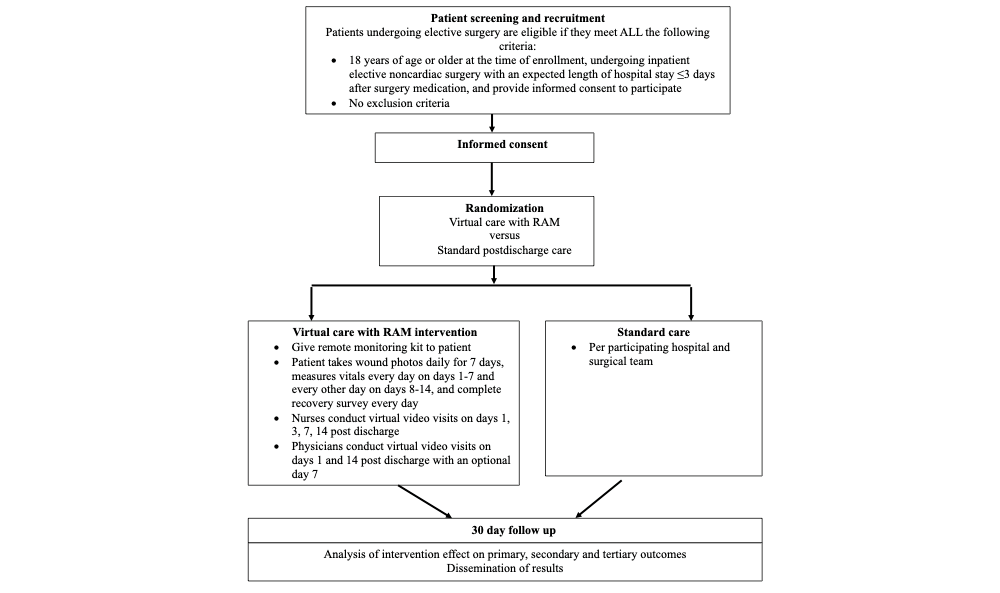

Supplement: Multimedia Appendix 1 [file jmir_v27i1e78893_app1.png]
